# Multiple Stones in a Single-System Ureterocele in a Child

**Published:** 2015-05-01

**Authors:** Sevgi Buyukbese Sarsu, Naim Koku, Suleyman Cuneyt Karakus

**Affiliations:** Department of Pediatric Surgery, Gaziantep Children’s Hospital, Gaziantep, Turkey

**Keywords:** Ureterocele, Urolithiasis, Hematuria

## Abstract

Presence of multiple calculi in ureterocele is rare in children. A 6-year-old boy presented with hematuria in whom on x-ray and ultrasound multiple calculi were noted in the urinary bladder. At surgery a ureterocele containing multiple calculi was found. The postoperative (99m) Tc-Dimercaptosuccinic acid scan (DMSA) reported normal renal function.

## INTRODUCTION

Ureterocele is an abnormal congenital dilatation of the submucosal part of ureter in the bladder. It may present in a single though more commonly observed with the duplex urinary system, in which case it is usually associated with upper moiety. Ureteroceles may cause complications ranging from urinary tract infections to renal scarring and upper pole destruction in the duplex systems. Stones in ureteroceles are less common in the pediatric population.[1] We present a 6-year-old boy with multiple stones in the right ureterocele diagnosed intra-operatively.

## CASE REPORT

A 6-years-old boy was admitted with hematuria. There was no previous history of urinary tract infection, hematuria, or abdominal pain. The physical examination was unremarkable and the laboratory tests were reported as normal except for hematuria. A plain abdominal radiograph demonstrated irregular radio opaque shadows in the pelvic area (Fig. 1). Abdominal ultrasonography showed a 3 cm × 2 cm, mobile, echoic area with an acoustic shadow in the bladder. Bladder wall thickness was 5 mm. No pathology was observed in the kidneys. Open surgery was performed based on the provisional diagnosis of urinary bladder stones; however, no stones were detected in it. On the right side a ureterocele was found. The edematous and hyperemic right ureterocele had an extremely irregular mucosal surface. A ureteral catheter was placed inside the left ureter, and then the roof of the catheterized right ureterocele was opened with a small incision in order to remove seven stones from the ureterocele (Fig. 2). Upon analysis of the stones, the composition was calcium oxalate (100%). The postoperative DMSA renal scan was normal. At 2-year follow-up, the patient is asymptomatic with no vesicoureteral reflux or stone recurrence.

**Figure:**
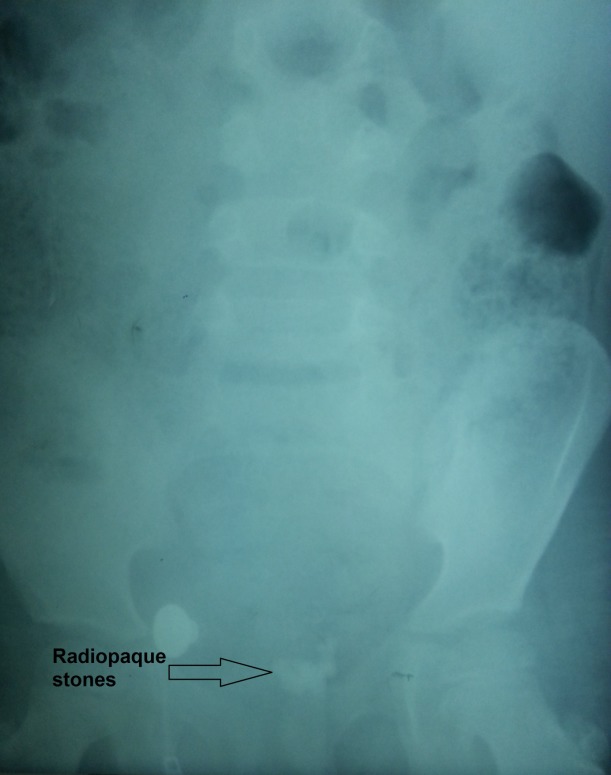
Figure 1: Plain abdominal radiograph showing radiopaque stones in the pelvis

**Figure:**
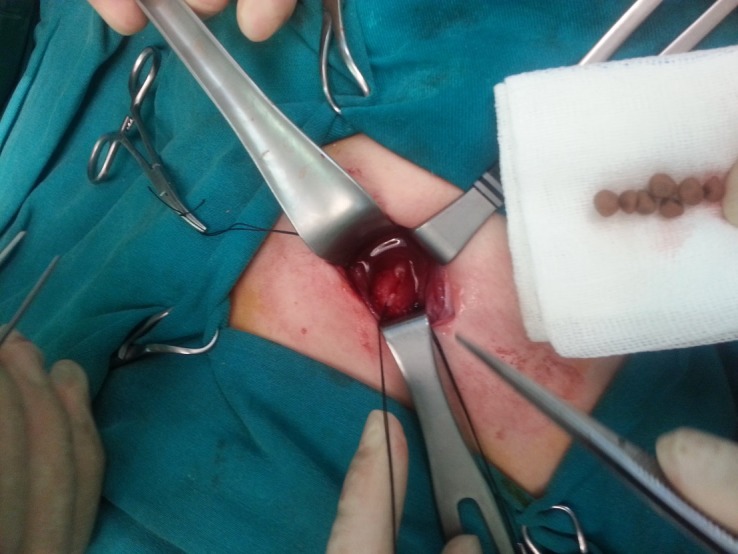
Figure 2: Removed seven stones from the ureterocele

## DISCUSSION

The presence of a single stone in a single ureterocele is often reported in adults in 4–39% cases though it is rarely reported in children.[4-6]. The important factors that predispose to stone formation in the ureterocele are ureteral atony, occlusion, urine stasis in the ureterocele, family history and a urinary tract infection.[2, 7] The stones in ureterocele remain asymptomatic and progressively grow, unless they lead to hematuria or an obstruction. Gilbert et al reported three stones in the left ureterocele in a child; a single stone in the ureterocele was reported in other three pediatric cases reported in literature.[4-6] In the index case, seven stones were removed from the right ureterocele.

Ultrasound and intravenous pyelogram are diagnostic in 50–70% cases.[3] Extremely irregular and edematous wall and the adequate opening of the ureterocele precluded our radiologist to diagnose it on ultrasound. However, the inability to detect the ureterocele with multiple stones may also be attributed to the radiologist’s inexperience. The aim of ureterocele treatment is to prevent urinary tract infections and vesicoureteral reflux while preserving renal function. Transurethral incision of the ureterocele and subsequent removal of the stones are the most commonly suggested treatment options for adult patients; excision of the ureterocele with either open or endoscopic surgery may also be preferred.[3,8] In conclusion, hematuria may be the initial presentation of a ureterocele with urolithiasis in children. Moreover, in cases with a diagnosis of bladder stones, the possibility of stones inside the ureterocele should be considered.

## Footnotes

**Source of Support:** Nil

**Conflict of Interest:** None declared

